# New understandings of the genetic regulatory relationship between non-coding RNAs and m^6^A modification

**DOI:** 10.3389/fgene.2023.1270983

**Published:** 2023-12-06

**Authors:** Songtao Liu, Dayong Xiang

**Affiliations:** ^1^ The First School of Clinical Medicine, Southern Medical University, Guangzhou, China; ^2^ Division of Orthopaedics and Traumatology, Department of Orthopaedics, Nanfang Hospital, Southern Medical University, Guangzhou, China; ^3^ Guangdong Provincial Key Laboratory of Bone and Cartilage Regenerative Medicine, Nanfang Hospital, Southern Medical University, Guangzhou, China

**Keywords:** biomarker, cancer, genetic regulation, m^6^A modification, non-coding RNA

## Abstract

One of the most frequent epigenetic modifications of RNA in eukaryotes is N6 methyladenosine (m^6^A), which is mostly present in messenger RNAs. Through the influence of several RNA processing stages, m^6^A modification is a crucial approach for controlling gene expression, especially in cancer progression. It is universally acknowledged that numerous non-coding RNAs (ncRNAs), such as microRNAs, circular RNAs, long non-coding RNAs, and piRNAs, are also significantly affected by m^6^A modification, and the complex genetic regulatory relationship between m^6^A and ncRNAs plays a pivotal role in the development of cancer. The connection between m^6^A modifications and ncRNAs offers an opportunity to explore the oncogene potential regulatory mechanisms and suggests that m^6^A modifications and ncRNAs could be vital biomarkers for multiple cancers. In this review, we discuss the mechanisms of interaction between m^6^A methylation and ncRNAs in cancer, and we also summarize diagnostic and prognostic biomarkers for clinical cancer detection. Furthermore, our article includes some methodologies for identifying m^6^A sites when assessing biomarker potential.

## Introduction

In messenger RNAs (mRNAs) or non-coding RNAs (ncRNAs), the sixth N atom of adenine A) gets methylated to form the m^6^A modification (Ping et al., 2014). With an average of around 3-5 m^6^A sites per mRNA, m^6^A modifications are the most prevalent and significant mRNA alteration in mammals, accounting for over 50% of all methyl-labeled RNAs (Wei et al., 1975), 0.1%–0.4% of all adenosine among all cellular RNAs, and an average of 3-5 m^6^A sites per mRNA (Rottman et al., 1974). The m^6^A modifications are particularly prevalent in the 3′-UTR, the stop codon, and the long internal exon in the mRNAs and frequently occur in the generally observed order RRACH (where R: A or G and H: A, C or U) (Fu et al., 2014). The splicing, export, translation, and degradation of mRNAs are all significantly impacted by these modifications (Wei and He, 2021). By regulating gene expression, m^6^A-modified RNAs influence a broad range of pathological and physiological processes (Frye et al., 2018). The occurrence and growth of cancer are highly correlated with abnormalities in m^6^A modification (He et al., 2019).

MicroRNAs (miRNAs), circular RNAs (circRNAs), and long non-coding RNAs (lncRNAs) have all been identified to have specific patterns that include the m^6^A modification, thanks to advancements in high-throughput genetic sequencing and mathematical techniques (Coker et al., 2019). Over ninety percent of the genome of mammalian cells is thought to be transcribed by ncRNAs, according to one research (Robinson et al., 2020). These ncRNAs operate as regulatory molecules that mediate various kinds of genetics activities, such as signal transduction, transcription, post-transcriptional changes, and chromatin remodeling. Therefore, the m^6^A modification of ncRNAs with modulatory capabilities is an instrumental element of epigenetic regulation. By altering the structure, biogenesis, and function of ncRNAs, m^6^A modifications impact diverse dimensions of pathophysiologic events (Huang et al., 2020). Dysregulation of the relationship between m^6^A modifying complements and ncRNAs has been linked to a variety of studies that have shown how cancer processes, including growth, aggression, and drug susceptibility, are affected (Ma et al., 2017; Li et al., 2019). This reveals a complicated web of prospective relationships connecting m^6^A and ncRNAs in the formation of cancer, whereby m^6^A and ncRNAs control target mRNAs by competing, cooperating, or both.

Despite being in its early stages, an abundant amount of dedication has gone into understanding the interaction between m^6^A regulators and non-coding RNAs. In this review, we summarize recent advances in the oncological field through our knowledge of the interactions between m^6^A and ncRNAs, concentrating on the potential regulatory mechanisms and biological changes of m^6^A-modified ncRNAs, together with the influence of ncRNAs on m^6^A regulators. Finally, we have generalized some diagnostic and prognostic biomarkers as well as methods for identifying the m^6^A sites, hoping that this will help in the detection and treatment of cancer.

## M^6^A regulators: writers, erasers and readers

**FIGURE 1:**
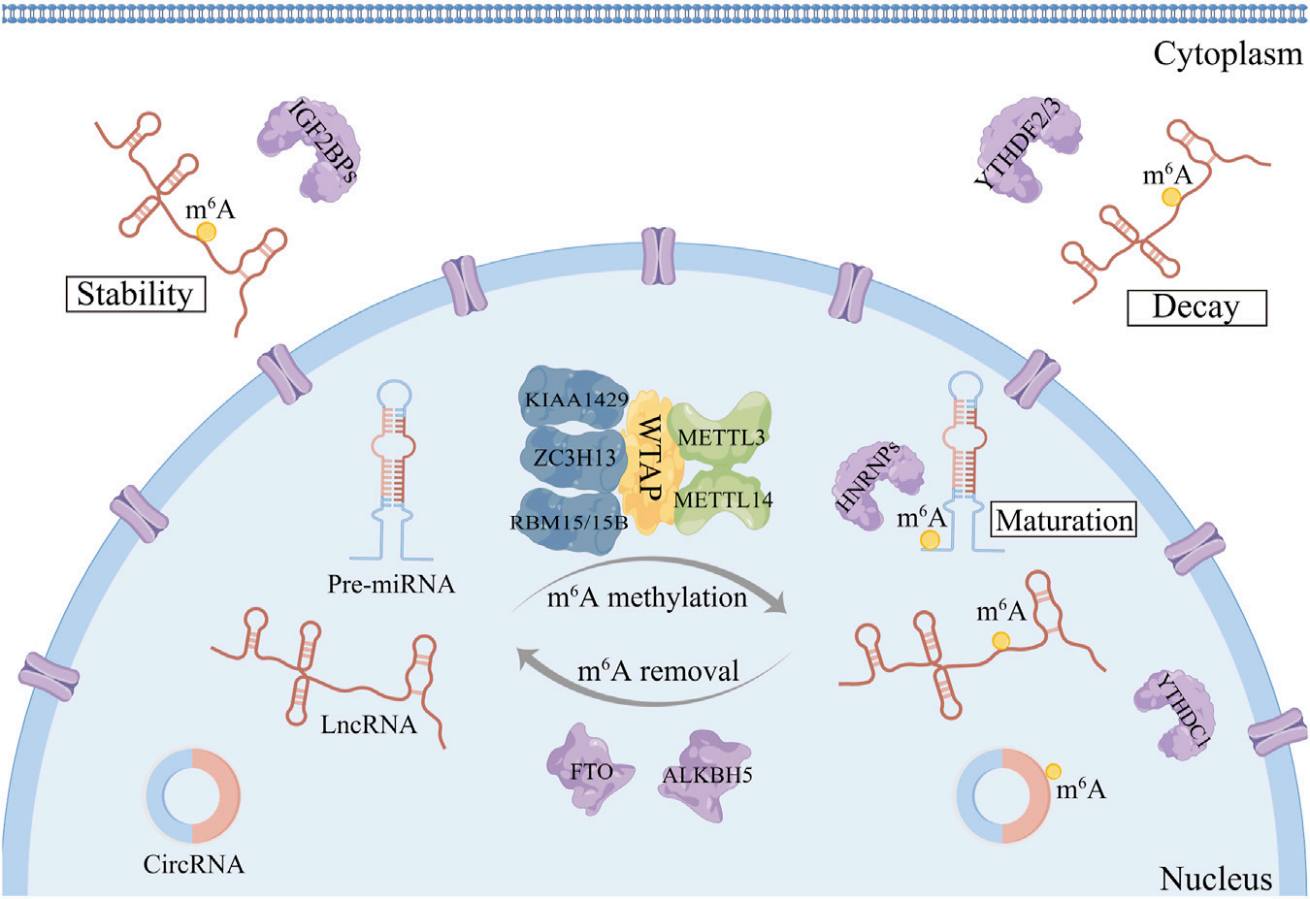
The dynamic and reversible process of m^6^A modifications on ncRNAs. M^6^A modifications are catalyzed by a writer complex consisting of METTL3-METTL14-WTAP core components as well as their cofactors KIAA1429 and RBM15/15B. The removal of m^6^A modifications depends on m^6^A erasers: FTO and ALKBH5. The function of m^6^A is mediated partly by m^6^A methylation recognition proteins, also called m^6^A readers, such as YTHDF1-3, YTHDC1-2, IGF2BP1-3, and HNRNPA2B1.

M^6^A-related proteins, which are also known as “writers,” “erasers,” and “readers,” are composed of the following components: m^6^A methyltransferases, m^6^A demethylases, and m^6^A identification factors (Figure 1). They have the capacity to add, delete or selectively identified m^6^A locations on non-coding RNAs respectively, thus altering important biological functions and affecting cancer development and progression (Shi et al., 2019).

### m^6^A writers

The methyltransferase complex (MTC), which consists of the proteins methyltransferase-like 3 (METTL3) (Chai et al., 2021), methyltransferase-like 14 (METTL14) (Wang et al., 2014), KIAA1429 (Jiang et al., 2021), Wilms tumor 1 associated protein (WTAP) (Jia et al., 2020), RNA-binding motif protein 15/15B (RBM15/15B) (Patil et al., 2016), and zinc finger CCCH structural domain-containing protein 13 (ZC3H13) (Wen et al., 2018), catalyzes m^6^A. As the catalytic core, the methyltransferase structural domain of METTL3 has catalytic activity (Wang et al., 2016). In addition to stabilizing MTC, METTL14 also recognizes certain RNA structures as catalytic substrates. In order to catalyze m^6^A methyltransferase, WTAP ligates METTL3/14 to create a complex that attaches to the nucleus (Schöller et al., 2018). In contrast, RBM15/15B recruits METTL3/14 complexes through WTAP-dependent methylation of U-rich sites (Yue et al., 2018), while KIAA1499 plays a clear role in the methylation deposition of the 3′-UTR (Knuckles et al., 2018). Meanwhile, ZC3H13 drives “writers” to the nucleus (Knuckles et al., 2018). However, it is still obscure how precisely these proteins regulate MTC development. It is also important to note that further research is still needed to determine if these methyltransferases may be employed as possible diagnostic biomarkers for cancer.

### m^6^A erasers

M^6^A methylation is a dynamic and reversible RNA alteration that may be removed by m^6^A demethylases, which are primarily composed of the proteins fat mass and obesity-associated protein (FTO) and alkB homolog 5 (ALKBH5) (Jia et al., 2011; Zheng et al., 2013). FTO and ALKBH5 are both members of the family of AlkB dioxygenases that remove m^6^A modifications in the presence of Fe(III) and 2OG, which is reliant on Fe(II) and 2-oxoglutarate (2OG) (Kurowski et al., 2003; Gerken et al., 2007). Labeled m^6^A annotations are removed from mRNAs and ncRNAs by the demethylation features included in FTO and ALKBH5. They encourage the demethylation response, which is crucial for the dynamic homeostasis of the m^6^A regulatory network, and they lower the degree of m^6^A modification in cellular mRNAs and ncRNAs. Notably, FTO possesses multiple substrates, including tRNA/U RNAs (Wei et al., 2018) and RNAs transcribed from repetitive elements (Wei et al., 2022). The functions of m^6^A writers and erasers as oncogenes and tumor suppressors in cancer have been established by several investigations. Mutations or dysregulation of m^6^A erasers are intrinsically linked to human cancer. Significant and valid RNA transcripts may experience aberrant m^6^A modifications due to instability brought on by the writer alone, the eraser alone, or both. These changes play a crucial part in the formation and advancement of tumors. However, the exact mechanisms regarding m^6^A erasers in cancer are less studied. Moreover, it may be worthwhile to investigate the possibility of more erasers in the future.

### m^6^A readers

The m^6^A regulatory system features a unique RNA-binding protein termed the m^6^A reader, in addition to writers and erasers. The reader is composed of the YT521-B homology (YTH) structural domain family (YTHDF1/2/3) (Shi et al., 2019), YTH structural domain-containing proteins (YTHDC1/2) (Haussmann et al., 2016), the heterogeneous nuclear ribonucleoprotein (HNRNP) protein family and insulin-like growth factor-2 mRNA-binding protein 1/2/3 (IGF2BP1/2/3, also known as IMP1/2/3) (Huang et al., 2018). Readers coordinate m^6^A-dependent control of RNA processing and performance, whereas writers and erasers collaborate to establish the spatial organization of m^6^A modifications on RNA. YTHDF1 and YTHDF2 increase the synthesis and degradation of m^6^A methylated mRNAs in the cytoplasm, respectively. On the contrary, YTHDF3 can help YTHDF2 accelerate the degradation of m^6^A-modified RNAs or promote m^6^A-modified RNA translation together with YTHDF1 (Li et al., 2017). YTHDC1 performs a variety of tasks, mostly in the nucleus, including boosting mRNA export, accelerating the degradation of particular transcripts, and coordinating mRNA splicing via the optimal assembly for particular splicing factors (Xiao et al., 2016). On the other hand, YTHDC2 is associated with the maintenance of mRNA and synthesis (Hsu et al., 2017). The most prevalent member of the HNRNP family that identifies m^6^A modification sites on specific primary miRNAs and comes into contact with drosha and DGCR8 to enhance pri-miRNA processing is HNRNPA2/B1 (Alarcón et al., 2015). To improve the integrity of mRNA and synthesis efficiency, IGF2BPs detect m^6^A modifications as well (Huang et al., 2018). Readers serve an essential regulatory function in how genes are expressed in the whole genome and significantly influence the formation of tumors or typical physiological processes (Chen et al., 2019). Because m^6^A needs to be recognized by readers to perform its corresponding function, inhibiting reader production or preventing reader identification of m^6^A can present a novel approach for cancer therapy.

## Roles of m^6^A regulators on non-coding RNAs in cancer

### MicroRNAs

MicroRNAs (miRNAs) are a category of short endogenous non-coding RNA transcript that ranges in length from 18 to 25 nucleotides (Saliminejad et al., 2019). MiRNAs attach to the 3′-untranslated region (3′UTR) of specific mRNAs, causing the breakdown of mRNA or inhibiting mRNA translation (Guajardo et al., 2020). There is emerging evidence that miRNAs possess oncogenic or antitumor capabilities by repressing tumor suppressors and oncogenes and are engaged in a broad range of biological and pathological events in human cancer (Van Roosbroeck and Calin, 2017). A great deal of researches have additionally concentrated on miRNA modifications in cellular malignant transformation and tumor progression, in which m^6^A is implicated.

### M^6^A regulators promote miRNAs maturation

Three phases make up the miRNAs biological genesis. Transcription is the initial step, during which miRNAs are converted into pri-miRNAs in the nucleus. Second, pri-miRNAs are transformed into pre-miRNAs with the aid of DGCR8 and Drosha. Pre-miRNAs are eventually transported to the cytoplasm for subsequent processing. Pre-miRNAs have been cleaved by Dicer to originate mature miRNAs in the cytoplasm (Mirihana Arachchilage et al., 2015). According to research, miRNA RNA maturation requires the m6A modification (Tang et al., 2021). To aid in miRNA maturation, METTL3 collaborates with the microprocessor protein DGCR8. Wen Peng et al. found that over-expression of METTL3 encourages pri-miR-1246 to mature and boosts the amount of miR-1246 in the body. Colorectal cancer cells migrate and invade when the oncogene SPRED2 is expressed less, as a result of miR-1246 (Peng et al., 2019). In pancreatic cancer, a high level of miR-380-3p expression is maintained by m^6^A modifications mediated by METTL3 and METTL14, which upregulate PTEN degradation and activate the Akt pathway, promoting tumor development (Jiang et al., 2022). Similar to this, METTL3 may boost miR-143-3p splicing in lung cancer (LC) cells to create more mature miRNAs and encourage lung cancer brain metastasis (Wang et al., 2019a). Interestingly, Rucheng Yan et al. found that bee toxin-induced selective METTL3 inhibition led to a reduction of miR-146a-5p, which ultimately activated the NOTCH2 pathway and induced apoptosis in bladder cancer (BC) cells (Yan et al., 2022). Deoxycholic acid, which binds to METTL3, inhibits the METTL3-METTL14-WTAP complex, according to Ruing Lin et al. This results in a reduction in miR92b-3p by interfering with the methylation of pri-miR-92b (Lin et al., 2020). These methylation-associated tumor suppressors provide innovative viewpoints on cancer therapy and may develop into a novel approach to the management of cancers.

M^6^A readers also facilitate the maturation of miRNAs. According to some reports, HNRNPA2B1 functions as an m6A reader that binds to the primary miRNAs and influences how miRNAs are processed. In lung adenocarcinoma (LUAD), HNRNPA2B1 reads the m6A region on pri-miR-106b to promote the developmental stage of miR-106b-5p, which suppresses SFRP2 and stimulates Wnt-β/catenin, thereby promoting cell stemness and LUAD progression (Rong et al., 2022). The processing of miRNAs in myeloma cells is also mediated by HNRNPA2B1, according to research by Rui Liu et al. The HNRNPA2B1-DGCR8 complex attaches to either pri-miR-92a-2-5p or pri-miR-373-3p, which promotes the maturation of miRNAs and upregulates their expression. Subsequently, both microRNAs are then transported via exosomes to recipient monocytes or mesenchymal stem cells, which activate osteoclastogenesis and inhibit osteoblastogenesis by blocking IRF8 or RUNX2 thereby causing bone damage (Liu et al., 2022a). It has been shown that YTHDC1 works with different pathways to control the expression of miRNAs. By opposing MCPIP1-mediated termination of miRNA biogenesis, YTHDC1 may facilitate the degradation associated with pri-miR-30d, followed by subsequent processing of mature miRNAs in pancreatic ductal adenocarcinoma. MiR-30d regulates the expression of SLC2A1 and HK1 by specifically aiming at RUNX1, which binds to the promoters of SLC2A1 and HK1 genes and thus inhibits aerobic glycolysis (Hou et al., 2021).

Taken together, we can find that the reader selectively recognizes the m^6^A motifs labeled by the writer and influences the interaction with DGCR8 to increase the output of pri-miRNAs through a microprocessor mechanism. The absence of m^6^A readers or m^6^A writers slows down the processing of the pri-miRNAs. It has been known for a long time that miRNAs exert their biological functions through direct binding to the 3′UTR of mRNAs. Studies have shown that there is a large enrichment of m^6^A peaks at the 3′UTR of mRNAs, and it is conceivable that the proximity of m^6^A to miRNA binding sites affects the miRNA-mediated mechanism of transcriptional repression (Meyer et al., 2012). The specific distribution of m6A may be a way to regulate the biological function of miRNAs.

### M^6^A regulators modify miRNAs to alter drug sensitivity

Sun et al. discovered that METTL3 boosts pri-miR-17-92 processing by modifying the A879 site via m^6^A modification. As an oncogenic miRNA cluster, the miR-17–92 cluster activates the AKT/mTOR pathway by inhibiting PTEN and TMEM127 to enhance GC development and metastasis. It is worth noting that this mechanism also increases the susceptibility of GC to treatment with everolimu (Sun et al., 2020a). Most of the studies were instead associated with increased cancer drug resistance.

### M^6^A regulators affect the miRNAs feedback loops

The m^6^A eraser can modulate the feedback loop of molecules. Some studies have shown that m^6^A erasers can function both directly within molecular feedback loops and indirectly outside of molecular feedback loops. ALKBH5 and miR-193a-3p form a positive feedback loop in which miR-193a-3p downregulates ALKBH5 expression, while ALKBH5 can prevent DGCR8 from recognizing precursor miR-193a-3p and the activation of mature miR-193a-3p. As miR-193a-3p increases, its mRNA degradation of ALKBH5 is enhanced, and the inhibitory effect of ALKBH5 on miR-193a-3p is weakened, which ultimately creates a positive feedback loop resulting in the accumulation of miR-193a-3p. A positive feedback loop between miR-193a-3p and ALKBH5 promotes ESCC growth and metastasis (Xue et al., 2021). Comparatively, FTO may encourage the proliferation of glioma cells by upregulating the levels of MYC, miR-155, and miR-23 through the MYC-miR-155/23a cluster-MXI1 positive feedback loop (Xiao et al., 2020). To be specific, as a demethylase, FTO can strengthen the stability of MYC mRNA by decreasing its m^6^A level. Overexpression of MYC significantly raised the levels of pri-miR-155 and pri-miR-23a, leading to increased miR-155 and miR-23a production. The tumor suppressor MXI1 was supposed to inhibit MYC levels by binding to the promoter of MYC; however, a large amount of miR-155 and miR-23a binds to the 3′UTR of MXI1, causing a decrease in its expression. In this way, MYC continues to accumulate, eventually leading to the proliferation of gliomas.

### Long non-coding RNAs

A family of lengthy transcripts known as long non-coding RNAs (lncRNAs) has in excess of 200 nucleotides and either no or minimal potential to code for proteins. The human genome contains several lncRNAs that are broadly transcribed (Deniz and Erman, 2017). The majority of research have discovered the biological functions of lncRNAs, which include the functionality of proteins, RNA equilibrium, and transcription modulation (Statello et al., 2021). An increasing body of research suggests that lncRNAs are crucial in regulating various cellular functions connected to cancer, including cell growth, invasion, migration, cell death, and stemness (Wang et al., 2020a; Luo et al., 2020; Monteiro et al., 2021).

### M^6^A regulators control the stability of lncRNAs

Multiple research investigations have demonstrated that m^6^A modifications possess a substantial impact on the long-term stability of ncRNAs, particularly lncRNAs. In head and neck cell carcinoma (HNSCC), METTL3 and METTL14-mediated m^6^A modifications to enhance the stability of LNCAROD in HNSCC cells (Ban et al., 2020). Not only that, in gastric cancer (GC), KIAA1429 overexpression promotes LINC00958 RNA residue levels and KIAA1429 regulates LINC00958 enrichment, which accelerates aerobic glycolysis in GC cells (Yang et al., 2021a). A substantial m^6^A modification site was discovered on FOXD2-AS1 by Zhipeng Ren et al. WTAP boosted the methylation modification, which improved the long-term viability of FOXD2-AS1 transcripts (Ren et al., 2022). Notably, the m^6^A writer not only enhances the stability of lncRNAs but also decreases it. METTL14 was found to downregulate XIST expression, thereby inhibiting CRC proliferation and metastasis (Yang et al., 2020). This suggests that the m^6^A writer can affect the stability of lncRNAs by altering the methylation level of lncRNAs.

M^6^A erasers affect the stability of lncRNAs in a variety of ways. Erasers can both improve the stability of ncRNAs, thereby upregulating their expression, and accelerate their degradation, thereby downregulating their expression. This seems to be related to the fact that lncRNAs can bind both different m^6^A readers and RNA-binding proteins. Research demonstrated that increased FTO in esophageal squamous cell carcinoma (ESCC) lowered the m6A methylation of LINC00022 transcripts, causing LINC000222 degradation to be repressed by YTHDF2. FTO overexpression has been found to promote LINC00022-dependent cell growth and tumor development (Cui et al., 2021). However, in hepatocellular carcinoma, ALKBH5 downregulates the lncRNA LINC02551, which is associated with the function of the demethyltransferase of ALKBH5 (Zhang et al., 2022a).

Numerous investigations have revealed that the effects of m^6^A methylation on RNA fate and function are mainly controlled through readers, and m^6^A readers may make the greatest contribution to the regulation of ncRNA stability. Senxu Lu et al. discovered that IMP2 functioned as a reader for m^6^A-modified ZFAS1 and enhanced the stability of lncRNA ZFAS1. Stable ZFAS1 binds to the OBG-type functional domain of OLA1, exposing the ATP binding site and increasing protein activity, eventually accelerating ATP hydrolysis and the Warburg effect in colorectal cancer (Lu et al., 2021). On the other hand, increasing m^6^A levels in the lncRNA TSUC7 and YTHDF2’s detection of the m^6^A peak in TSUCC7 led to the degradation of TSUC7, which made lung cancer more resistant to erlotinib (Li et al., 2021a). Notably, Hongmei Liu et al. discovered that YTHDF1 and YTHDF2 balance function in the transcription or degradation of lncRNA THOR by binding to different sites and thus in lncRNA THOR. Specifically, modifications of THOR sites 2 and 3 promoted LC cell proliferation, whereas modifications of THOR sites 4 and 5 inhibited lung cancer cell proliferation (Liu et al., 2020). Interestingly, YTHDF1 could adhere to m^6^A modification sites to stimulate lncRNA translation. It has been shown that the Y-linked lncRNA LINC00278 has the ability to encode the neuropeptide YY1BM. Additionally, LINC00278 contains a traditional m6A modification motif adjacent to the YY1BM stop codon, which interacts with YTHDF1 and facilitates the translation of YY1BM. By preventing the transcription of eEF2K, which enhances eEF2 function and causes ESCC apoptosis, YY1BM suppresses the connection between YY1 and AR (Wu et al., 2020).

### M^6^A regulators participate in the ceRNA mechanism associated with lncRNAs

M^6^A writers can regulate the endogenous competition mechanism of lncRNAs. According to an increasing number of research projects, lncRNAs may control the expression of miRNAs and hence affect the expression of downstream target mRNAs by acting as molecular sponges for miRNAs. This process is known as the ceRNA mechanism (Qi et al., 2020). The longevity of lncRNAs may be controlled by m^6^A modifications, which can also have an impact on the ceRNA machinery as previously mentioned (Li Z. et al., 2021). It has been demonstrated that LINC00958 is upregulated by METTL3-mediated N6 methyladenosine modification, which stabilizes its RNA transcripts. Furthermore, LINC00958 can uptake miR3619-5p to increase HDGF expression, which promotes adipogenesis and the progression of hepatocellular carcinoma (HCC) (Zuo et al., 2020). In addition to this, METTL3 maintains the upregulation of LNCAROD by increasing m^6^A methylation-mediated RNA stability, while LNCAROD increases PKM2 levels by simultaneously enhancing SRSF3-mediated PKM to PKM2 conversion and uptake of miR-145-5p to increase PKM2 levels, ultimately increasing aerobic glycolysis in HCC cells (Jia et al., 2021). In contrast, in cholangiocarcinoma, m6A-modified NKILA attenuates its inhibition of miR-582-3p (Zheng et al., 2022).

### M^6^A regulators modify lncRNAs to alter drug sensitivity

In breast cancer (BRC), WTAP binds to the m^6^A site of DLGAP1-AS1 and promotes its stability, while lncRNA DLGAP1-AS1 in turn increases its adriamycin resistance through the WTAP/DLGAP1-AS1/miR-299-3p feedback loop (Huang et al., 2021). Additionally, the MALAT1-miR-1914-3p-YAP axis is regulated by METTL3-initiated m^6^A mRNA methylation, which enhances YAP activity and directly stimulates YAP translation in non-small cell lung cancer (NSCLC) (Jin et al., 2021). This results in treatment resistance and metastasis. Previous research has also discovered that lncRNA ANRIL splicing, which is dependent on the m^6^A methylation of ANRIL, is how SRSF3 gene expression desensitizes tumor cells in the pancreas to the chemotherapy medication gemcitabine (Wang et al., 2022a). The influence of m^6^A writers on lncRNAs sheds light on the molecular processes behind cancer chemotherapy, defining a sound direction for advancing cancer therapies in the future.

The m^6^A erasers can also regulate cancer sensitivity to drugs. By controlling the degree of stability of lncRNAs, m^6^A erasers, like m^6^A writers, may affect how responsive cancer is to chemotherapy. Chengjie Lin et al. found that ALKBH5-mediated m^6^A demethylation promoted SH3BP5-AS1 degradation, which inhibited the Wnt signaling pathway and thus reduced chemoresistance to gemcitabine in pancreatic cancer (Lin et al., 2022a). In esophageal squamous cell carcinoma, ALKBH5-mediated m^6^A removal is linked to regulating the lncRNA CASC8, which enhances cell growth and cisplatin resistance in ESCC (Wu et al., 2022). Although current studies on the regulation of drug sensitivity by m^6^A erasers are still lacking, immunotherapy related to m^6^A erasers may offer new opportunities for cancer treatment (Yang et al., 2019).

### M^6^A regulators affect the lncRNAs feedback loops

M^6^A readers contribute to the creation of molecular negative feedback loops. Unlike m^6^A erasers that often regulate molecular positive feedback loops, m^6^A readers are involved in molecular negative feedback loops. Wen Ni et al. discovered that the LncRNA GAS5 binds straight to YAP, boosting its phosphorylation and ubiquitin-mediated degeneration and therefore impeding YAP-mediated production of YTHDF3. Whereas YTHDF3 attaches m6A-methylated GAS5 temporarily and preferentially to cause its decay and establish a negative feedback loop that slows CRC growth (Ni et al., 2019). Furthermore, it has been reported that a stronger relationship between the m^6^A reader YTHDF3 and DICER1-AS1 leads to DICER1-AS1 degeneration as a consequence of glucose exhaustion, and YTHDF3 has an essential focus of miR-5586-5p through which it can form a negative feedback loop with DICER1-AS1 to promote the process of glycolysis in pancreatic cancer (Hu et al., 2022). The specificity of the feedback loop provides new possibilities for the control of tumor malignant behavior.

### M^6^A regulators manipulate the local structure of lncRNAs

Recently, several studies have revealed that m^6^A regulators can promote the binding of lncRNAs to certain RNA-binding proteins by adjusting their local structures, ultimately affecting tumorigenesis and progression. For example, as a lncRNA highly expressed in the nucleus, MALAT1 modified by m^6^A can alter its localization and activity in the nucleus and bind to different regulatory proteins through changes in RNA structure. In the clip structure of MALAT1, there are some sites that can be modified by m^6^A. As the level of m^6^A modification increases, the U-A pairing effect will diminish, and the hairpin structure will become slack. This augments the binding of the lncRNA MALAT1 to m^6^A readers. To be specific, methylation of sites A2577 and A2515 in MALAT1 allows the surrounding RNA sequences to bind HNRNPC and HNRNPG more readily. Lowering of METTL3/METTL14 reduces the exposure of MALAT1 to these proteins, hence suppressing the proliferation of tumors (Liu et al., 2017).

### Circular RNAs

The production of circular RNAs (circRNAs), a novel class of endogenous RNA molecules that do not code with a covalent closed-loop structure, is predominantly accomplished by a process known as reverse splicing (Sanger et al., 1976). Circular RNAs (circRNAs) lack a 5′cap and a 3′poly(a) tail, therefore being chemically closed in contrast to normal linear RNAs (Grabowski et al., 1981), which makes them more resistant to nucleic acid exonucleases. CircRNAs are significant in several diseases, especially human cancer, because they are plentiful, durable, evolutionarily preserved, and have spatiotemporal-specific expression patterns (Memczak et al., 2013; Li et al., 2015). CircRNAs control many different processes, including collaborating with RNA-binding proteins, serving as sponges for microRNAs, controlling gene transcription, and modulating the translation of genes into amino acids (Chen, 2020).

### M^6^A regulators control the stability of circRNAs

In hepatocellular carcinoma, IGF2BP1 may attach to circMDK and enhances the transcriptome stability of circMDK. CircMDK uptake miR-346 and miR-874-3p to upregulated ATG16L1, which activates the PI3K/AKT/mTOR signaling pathway and promotes cell growth, migration as well as invasion (Du et al., 2022). M^6^A modifications in colorectal cancer are also present on circ3823, and the m6A recognition protein YTHDF3 and the demethylase ALKBH5 regulate the rate of degradation of circ3823. Degradation of circ3823 inhibits proliferation, metastasis, and angiogenesis of CRC cells (Guo et al., 2021). Additionally, by making circCCDC134 more stable via YTHDF2, which encourages cervical cancer (CC) spread, ALKBH5-mediated m6A methylation is in charge of its overexpression (Liang et al., 2022).

### M^6^A regulators participate in the ceRNA mechanism associated with circRNAs

CircRNAs may, like lncRNAs, operate as molecular sponges for miRNAs, which may enable them to control the synthesis of miRNAs and have an influence on the expression of downstream target mRNAs. Similarly, m^6^A regulators can modulate this process. In gastric cancer, silencing of METTL14 decreased m^6^A levels of circORC5 but increased circORC5 expression. circORC5 could take up miR-30c-2-3p and reverse METTL14-induced miR-30c-2/3p upregulation and AKT1S1 and EIF4B downregulation. This suggests that METTL14-mediated m^6^A modification of circORC5 inhibits GC progression by regulating the miR-30c2-3p/AKT1S1 axis (Fan et al., 2022). On the contrary, in colorectal cancer, METTL3-mediated m^6^A modification enhances the binding of circALG1 to miR-342-5p, thereby upregulating placental growth factor (PGF) expression, which accelerates cancer cell metastasis (Lin et al., 2022b).

### M^6^A regulators modify circRNAs to change radiation resistance

M^6^A writers can also increase cancer resistance to radiotherapy. In hypopharyngeal squamous cell cancer (HPSCC), Ping Wu et colleagues. observed that METTL3-mediated m^6^A modification preserved circCUX1 expression, suppressing caspase 1 production and imparting radiation resistance (Wu et al., 2021a).

### M^6^A regulators drive circRNA translation initiation

Previous studies have shown that, with the help of m^6^A regulators, some of the circRNAs can exhibit the ability to encode proteins. This is due to the fact that some of the m^6^A methylation sites in circRNAs can operate as internal ribosomal entry sites (IRESs) to drive protein translation (Tang et al., 2020). Specifically, the writers METTL3 and METTL14 facilitate the process of translation by raising the m^6^A methylation level of circRNAs; in contrast, the eraser FTO-mediated demethylation process hinders translation. As an m^6^A reader, YTHDF3 plays an equally pivotal role in this process (Yang et al., 2017). Translation of circRNAs under m^6^A modification opens new perspectives, which may become a significant breakthrough in research.

In conclusion, m^6^A regulators play important roles in several cancers by influencing different biological functions in ncRNAs (Table 1).

**TABLE 1:** m^6^A regulators modify ncRNAs in cancer.

Proteins	Related non-coding RNA	Cancer	Function	Role in cancer	Regulation	References
METTL3	miR-1246	CRC	Writer	Oncogene	Upregulated	Peng et al. (2019)
METTL3	miR-143-3p	LC	Writer	Oncogene	Upregulated	Wang et al. (2019a)
METTL3	MALAT1	LC	Writer	Oncogene	Upregulated	Jin et al. (2021)
METTL3	LNCAROD	HCC	Writer	Oncogene	Upregulated	Jia et al. (2021)
METTL3	circCUX1	HPSCC	Writer	Oncogene	Upregulated	Wu et al. (2021a)
METTL14	XIST	CRC	Writer	Tumor suppressor gene	Downregulated	Yang et al. (2020)
METTL14	circORC5	GC	Writer	Tumor suppressor gene	Downregulated	Fan et al. (2022)
WTAP	DLGAP1-AS1	BC	Writer	Oncogene	Upregulated	Huang et al. (2021)
KIAA1429	LINC00958	GC	Writer	Oncogene	Upregulated	Yang et al. (2021a)
ALKBH5	CirCCDC134	CC	Eraser	Oncogene	Upregulated	Liang et al. (2022)
FTO	LINC00022	ESCC	Eraser	Oncogene	Upregulated	Cui et al. (2021)
YTHDC1	miR-30d	PDAC	Reader	Tumor suppressor gene	Upregulated	Hou et al. (2021)
YTHDF1	LINC00278	ESCC	Reader	Tumor suppressor gene	Upregulated	Wu et al. (2020)
YTHDF2	TSUC7	LUAD	Reader	Oncogene	Downregulated	Li et al. (2021a)
IGF2BP1	circMDK	HCC	Reader	Oncogene	Upregulated	Du et al. (2022)
IMP2	ZFAS1	CRC	Reader	Oncogene	Upregulated	Lu et al. (2021)
HNRNPA2B1	miR-106b-5p	LUAD	Reader	Oncogene	Upregulated	Rong et al. (2022)

Abbreviation:CRC, colorectal cancer; LC, lung cancer; HCC, hepatocellular carcinoma; HPSCC, hypopharyngeal squamous cell carcinoma; GC, gastric cancer; BC, breast cancer; CC, cervical cancer; ESCC, esophageal squamous cell carcinoma; PDAC, pancreatic ductal adenocarcinoma; LUAD, lung adenocarcinoma.

## Roles of non-coding RNAs on m^6^A regulators in cancer

**FIGURE 2:**
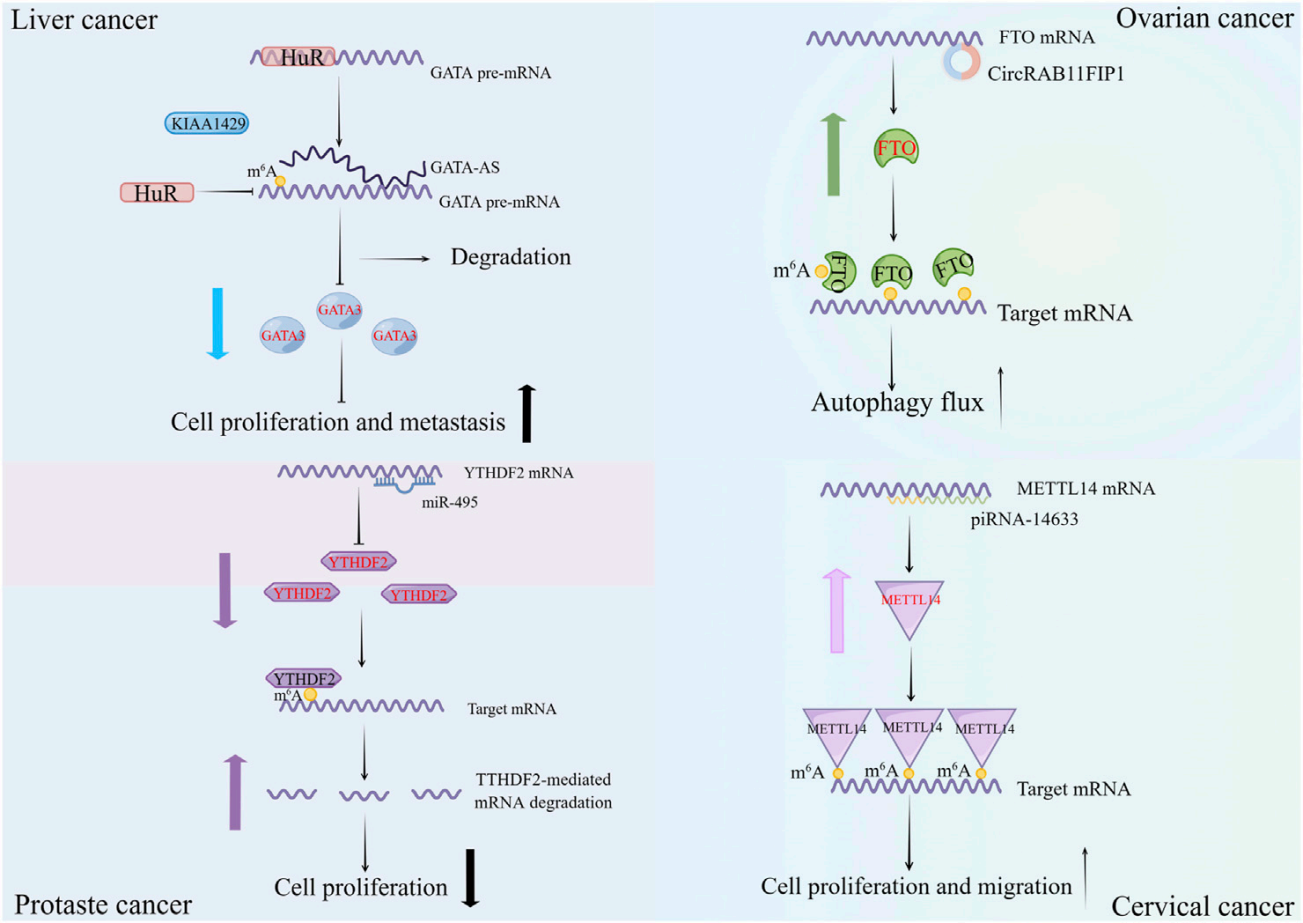
Examples of non-coding RNAs (lncRNA, circRNA, miRNA and piRNA) modulating m^6^A regulators. Different classes of non-coding RNAs can directly modulate the expression of m^6^A regulators or indirectly guide the function of m^6^A regulators to influence the malignant behavior of cancers.

With the advancement of ncRNAs and m^6^A regulators studies, more and more occurrences demonstrate that not only can m^6^A regulators impact ncRNAs, but ncRNAs may additionally influence the generation and activities of m^6^A regulators (Figure 2). Abnormal levels of ncRNAs can correspondingly lead to changes in m^6^A levels, which have an important role in cancer development.

### Roles of miRNAs on m^6^A regulators in cancer

MiRNAs downregulate the expression levels of m^6^A regulators. For m^6^A writers, it was shown that in NSCLC, miR-4443 modulates cisplatin resistance *in vivo* by means of METTL3/FSP1 pathway. MiR-4443 overexpression suppresses cisplatin-induced ferroptosis by negatively regulating METTL3-induced FSP1 m^6^A modification (Song et al., 2021). Interestingly, Liyan Wang et al. discovered that exosomes produced from M1 macrophages transported miR-628-5p to hepatocellular carcinoma cells to restrict METTL14 expression, which lowered the degree of circFUT8 m^6^A modification, hence reducing HCC growth (Wang et al., 2022b). For m^6^A readers, YTHDF2 molecule is downregulated by miR-6125, which boosts the degree of durability of GSK3 mRNA. MiR-6125 aims at the 3′-UTR of YTHDF2. In contrast, elevated GSK3 concentrations of protein restricts the production of components connected to the Wnt/-catenin/Cyclin D1 pathway, causing termination in the G0-G1 phase and ultimately suppressing CRC cell proliferation (Li et al., 2021c). Similarly, miR-495 has YTHDF2 as a target gene, which it may use to interfere with the production of YTHDF2 in prostate cancer cells. On the other hand, amplification of YTHDF2 might disrupt with miR-495 suppressive effects on growth, aggression, and movement of prostate cancer cells as well as its triggering of apoptosis (Du et al., 2020). In terms of m^6^A erasers, Jiazhu Sun et al. found that miR-5581-3p dramatically decreased FTO expression, which subsequently hindered BC cell lines proliferation and mobility (Sun et al., 2022). Notably, miRNAs could, in an indirect manner, upregulate the expression of m^6^A erasers. It has been shown that miR-96 may inhibit AMPK2, which increases the expression of FTO and, by preventing its m^6^A modification, upregulates the expression of MYC. The pro-proliferative and anti-apoptotic actions of miR-96 in colorectal cancer cells are also connected to this mechanism (Yue et al., 2020).

MiRNAs have an impact on the activity of m^6^A regulators. Besides binding to mRNAs to promote their degradation, miRNAs were found to recruit m^6^A erasers. In glioblastoma, miR-145 recruited FTO and induced the attachment of FTO and CLIP3 mRNA, and increased intracellular demethylase action, while the translation efficiency of m^6^A-deficient transcripts was significantly increased (Zepecki et al., 2021). Not only that, miRNAs can compete with each other for binding sites with m^6^A readers, thus inhibiting the translation of related proteins. For example, miR455-3p and METTL3 might contend for the sequence change of the HSF1 mRNA, which would prevent HSF1 protein synthesis. The HSF1 gene expression and pharmaceutical treatment both diminish the development of colorectal cancer in mice (Song et al., 2020). On the one hand, the creation of miRISC as a result of miRNA binding may obstruct interactions with METTL3. On the other hand, once mRNA is formed, the m^6^A modification modifies its molecular shape and construction, which has an effect on how miRNA and mRNA pair up (Roost et al., 2015). However, how exactly the binding of miRNA to mRNA interferes with the function of METTL3 still needs to be investigated.

### Roles of lncRNAs on m^6^A regulators in cancer

LncRNAs have the ability to recruit m^6^A regulators to control the metabolism of downstream molecules. Further research has revealed that lncRNAs may recruit m^6^A writers and readers to control the durability of downstream mRNAs. Regarding the regulation of m^6^A writers, it has been shown that LNC942 straightforwardly attracts METTL14 by carrying particular recognition sequences, thereby maintaining the expression of LNC942 downstream targets *in vitro* and *in vivo* through post-transcriptional m^6^A methylation modifications, which exerted a robust oncogenic effect in promoting breast cancer cell growth and development of colonies along with inhibiting apoptosis potent oncogenic effects (Sun et al., 2020b). The long non-coding RNA GATA3-AS, which is translated from the GATA3 gene’s antisense strand, promotes tumor development and metastasis in hepatocellular carcinoma by interacting with KIAA1429 and GATA3 pre-mRNA and instructing KIAA1422 to preferentially induce m^6^A modification of GATA3 pre-mRNA (Lan et al., 2019). METTL3 is reportedly brought in by ARHGAP5-AS1 to promote m^6^A modification of ARHGAP5 mRNA, which stabilizes ARHGAP5 in its cytoplasm as well. Because of this, ARHGAP5 is overexpressed to foster chemoresistance, and this overexpression is associated with a poor prognosis in GC (Zhu et al., 2019). According to Xinyu Wang et al.'s research on the regulation of m^6^A readers, m^6^A residues attached to MALAT1 served as a scaffold for drawing YTHDC1 to nuclear patches, largely enhancing the ability of esophageal cancer cells to spread (Wang et al., 2021a). By attaching to the m^6^A reader IGF2BP1, which is crucial to *in vivo* stemness and tumorigenesis in BCSCs, LncRNA KB-1980E6.3 enhances the endurance of the c-Myc mRNA in breast cancer (Zhu et al., 2021). Related studies have shown that NEAT1 prevents TRIM25-mediated ubiquitination of RPRD1B and lowers RPRD1B protein decomposition by attracting hnRNPA2B1 (Jia et al., 2022). In contrast, via the c-Jun/c-Fos/SREBP1 axis, RPRD1B boosts fatty acid consumption in gastric cancer and assists the initial tumor colonization in lymphatic system. Similarly, in the GC, lncRNA CBSLR recruits YTHDF2 to establish the CBSLR/YTHDF2/CBS signaling axis, which decreases the longevity of CBS by increasing YTHDF2 adhering to the m6A-modified coding sequence (CDS) of CBS mRNA. Patients with gastric cancer with a high CBSLR/low CBS level had inferior clinical outcomes and treatment responsiveness (Yang et al., 2022a).

LncRNAs enhance the stability of m^6^A regulators. The effect of lncRNAs on the stability of m^6^A regulators is mainly achieved by increasing the stability of m^6^A readers. By inhibiting IGF2BP2’s ubiquitination at K139 and shielding it from the autophagic lysosomal pathway’s (ALP) destruction, LINRIS preserves IGF2BP2’s stability. In CRC cells, MYC-mediated glycolysis is mainly affected by IGF2BP2’s downstream effects, which are attenuated when LINRIS is knocked down (Wang et al., 2019b). Similarly, LCAT1 attaches to IGF2BP2 and stops it from degrading in the lysosome. The cellular production of CDC6 is then upregulated by stabilized IGF2BP2 in a m^6^A-dependent mechanism. Lung cancer cells may multiply, remain alive, and migrate with greater efficiency when CDC6 accumulates (Yang et al., 2022b). In renal clear cell carcinoma, DMDRMR specifically enhances IGF2BP3 activity in a way associated with m^6^A modification, stimulating the G1-S transition and thus promoting cell proliferation (Gu et al., 2021). Specifically, Song Zhu et al. found that lncRNAs can also interact with m^6^A readers through their own translated peptides. The LINC00266-1 encodes a 71-amino acid peptide. Because this peptide primarily cooperates with RNA-binding proteins, such as the m^6^A reader IGF2BP1, it is known as an “RNA-binding regulatory peptide” (RBRP). RBRP attaches IGF2BP1 and promotes m^6^A identification of c-myc by IGF2BP1, increasing the lifespan of mRNA and up-regulating c-myc expression, facilitating carcinogenesis (Zhu et al., 2020). A handful of lncRNAs have been discovered to modulate m^6^A writers as well. The lncRNA UCA1, for example, accelerates the course of acute myeloid leukemia by connecting to METTL14 to elevate m6A quantities and augment CXCR4 and CYP1B1 expression (Li et al., 2022a). Nevertheless, the modulation of m^6^A erasers associated with lncRNAs has only occasionally been documented, which could offer fresh perspectives on the course of future research and open up opportunities for the treatment of cancer.

### Roles of circRNAs on m^6^A regulators in cancer

CircRNAs have the capacity to control how m^6^A regulators coordinate with downstream molecules. Numerous studies have shown that circRNAs can also recruit m^6^A readers to affect the activity of downstream mRNAs. According to research, circRHBDD1 increases the metabolic reprogramming of HCC and reduces the effectiveness of anti-PD-1 treatment by attaching YTHDF1 to PIK3R1 mRNA and accelerating PIK3R1 translation in a way that depends on m^6^A (Cai et al., 2022). In addition to this, in NONO-TFE3 fused renal cell carcinoma, circMET recruits YTHDF2 by directly binding CDKN2A mRNA, which significantly impairs CDKN2A expression and leads to CDKN2A mRNA attenuation (Yang et al., 2021b). Notably, circRNAs can also regulate mRNA stability by switching m^6^A readers. For instance, rtcisE2F hampers the binding of E2F6/E2F3 mRNAs with YTHDF2 and enhances their connection with IGF2BP2. E2F6/E2F3 mRNA decay is prevented by IGF2BP2, but E2F6/E3F3 mRNA decay is accelerated by YTHDF2. By converting the m^6^A reader, rtcisE2F increases the stability of E2F6/E2F3 mRNA, which promotes liver tumorigenesis and metastasis by triggering self-regeneration of liver tumor-initiating cells (Chen et al., 2022). CircRNAs also interfere with the recognition of m^6^A sites by m^6^A readers. Fei Xie et al. found that circPTPRA partially interfered with IGF2BP1-mediated recognition of m^6^A-modified RNAs through interaction with the KH structural domain of IGF2BP1. The interaction of circPTPRA with IGF2BP1 attenuates the regulation of MYC and FSCN1 by IGF2BP1, thereby suppressing the progression of BC (Xie et al., 2021). Similarly, circRNAs can regulate the role of m^6^A writers. In BC cells, it has been shown that circ0008399 associates with the WTAP protein to foster the development of the WTAP/METTL3/METTL14 compound. By boosting the mRNA stabilization of the intended gene TNFAIP3 in a m^6^A-dependent manner, Circ0008399/WTAP compound stimulates its expression (Wei et al., 2021). Furthermore, circ-CTNNB1 interacts with the writer RBM15, promoting the generation of HK2, GPI, and PGK1 via m^6^A modification to accelerate the glycolytic procedure and stimulate osteosarcoma advancement (Yang et al., 2023).

CircRNAs have the ability to influence the synthesis of m^6^A regulators. CircRNAs have a wide range of effects on the expression levels of m^6^A regulators, whether they are writers, erasers, or readers, which are regulated by it. According to a publication, circRERE regulates GBX2 expressing themselves in HCC via ZC3H13/m^6^A, and down-regulating circRERE significantly boosts ZC3H13 production (Lin et al., 2022c). Furthermore, circRAB11FIP1 connects to FTO as well as advocates its translation, which increases autophagy and malignant behavior in ovarian cancer (EOC), according to research by Zhanqin Zhang et al. (Zhang et al., 2021). CircNDUFB2 is a scaffold in NSCLC to strengthen the bond between TRIM25 and IGF2BPs. This ternary combination of TRIM25, circNDUFB2, and IGF2BPs fosters the ubiquitination and breakdown of IGF2BPs, eventually preventing NSCLC progress and dissemination (Li et al., 2021d). CircRNAs, in particular, may serve as miRNA sponges that indirectly modulate the production levels of m^6^A regulators. For instance, CircVMP1 functions as an absorbent for miR-524-5p and upregulates the levels of METTL3 and SOX2, enhancing the growth of NSCLC and cisplatin resistance (Xie et al., 2022a). According to Lianyong Liu et al., circGPR137B, which shared a location with miR-4739 in the cytoplasm, served as a sponge for miR-4739 and upgraded the level of its target FTO, inhibiting cell proliferation and cancer metastasis in HCC patients. Patients who had high levels of circGPR137B expression also had better survival rates (Liu et al., 2022b). It has been observed that circEZH2 works as a sponge for miR-133b, causing IGF2BP2 to be upregulated in addition to its association with the IGF2BP2 and blocking its ubiquitination-dependent destruction. CircEZH2/IGF2BP2 facilitates the advancement of CRC by boosting CREB1 mRNA stability (Yao et al., 2022).

### Piwi-interacting RNA

A tiny non-coding RNA known as piwi-interacting RNA (piRNA) is described as binding selectively to Piwi family proteins in testicular germ cells (Rayford et al., 2021). It regulates mRNA turnover and also directs transposon silencing by regulating DNA methylation (Wang et al., 2021b). Evidence from recent studies has shown that piRNAs are effectively generated in both natural tissue cells and tumor cells (Liu et al., 2019). Additionally, PiRNA has been demonstrated to possess pro- or anti-cancer effects by simply attaching to RNA-binding proteins called PIWI proteins (Zeng et al., 2020). As piRNA was progressively explored, it was found that it could affect a variety of processes in cancer cells by controlling m^6^A regulators gene expression.

### Roles of piRNAs on m^6^A regulators in cancer

PiRNAs upregulate the expression levels of m^6^A regulators. It has been shown that piRNA accumulation has concentration-dependent effects on the production of METTL14 in cervical carcinoma. PiRNA-14633 mimics elevated levels of m^6^A RNA methylation and stabilized METTL14 mRNA, which eventually aided in the formation of cervical cancers (Xie et al., 2022b). Similar to this, piRNA-30473 triggers m^6^A methylation in diffuse large B-cell lymphoma (DLBCL) via enhancing WTAP production. In particular, by adhering to the 3′UTR of WTAP, piRNA-30473 decreases WTAP mRNA degradation and ultimately improves mRNA balance, thus prompting WTAP to perform an oncogenic function in DLBCL(134). Moreover, piRNA-17560, an exosome generated by senescent neutrophils, increases the replication of FTO in breast tumor cells. Augmentation of FTO facilitates transition from epithelial to mesenchymal and chemotherapy resistance in cancer cells by lowering m^6^A levels and improving ZEB1 transcript stabilization and activation (Ou et al., 2022). Unfortunately, studies on piRNAs have all been very scarce in recent years, and further investigation of piRNAs is still needed. In addition, whether there are other modalities of regulation of m^6^A regulators by piRNAs is also a direction that needs to be explored in the future.

Taken together, these discoveries suggest that ncRNAs can impact m^6^A modifications on mRNAs in terms of their expression and function by targeting m^6^A writers, erasers, and readers (Table 2), hence offering a basis for future investigation of the role of RNA epigenetic modulation mechanisms in cancers.

**TABLE 2:** ncRNAs modulate m^6^A regulators in cancers.

Related non-coding RNA	Proteins	Cancer	Function	Role in cancer	Regulation	References
UCA1	METTL14	AML	Writers	oncogene	Upregulated	Li et al. (2022a)
LINC00266-1	IGF2BP1	CRC	Readers	oncogene	Upregulated	Zhu et al. (2020)
LINRIS	IGF2BP2	CRC	Readers	oncogene	Upregulated	Wang et al. (2019b)
LCAT1	IGF2BP2	LC	Readers	oncogene	Upregulated	Yang et al. (2022b)
DMDRMR	IGF2BP3	ccRCC	Readers	oncogene	Upregulated	Gu et al. (2021)
circRERE	ZC3H13	HCC	Writers	oncogene	Upregulated	Lin et al. (2022c)
circRAB11FIP1	FTO	ETC	Erasers	oncogene	Upregulated	Zhang et al. (2021)
circEZH2	IGF2BP2	CRC	Readers	oncogene	Upregulated	Yao et al. (2022)
miR-4443	METTL3	NSCLC	Writers	oncogene	Downregulated	Song et al. (2021)
miR-628-5p	METTL14	HCC	Writers	Tumor suppressor gene	Downregulated	Wang et al. (2022b)
miR-5581-3p	FTO	BCa	Erasers	Tumor suppressor gene	Downregulated	Sun et al. (2022)
miR-96	FTO	CRC	Erasers	oncogene	Upregulated	Yue et al. (2020)
miR-6125	YTHDF2	CRC	Readers	Tumor suppressor gene	Downregulated	Li et al. (2021c)
miR-495	YTHDF2	PCa	Readers	Tumor suppressor gene	Downregulated	Du et al. (2020)
piRNA-14633	METTL14	CC	Writers	oncogene	Upregulated	Xie et al. (2022b)
piRNA-30473	WTAP	DLBCL	Writers	oncogene	Upregulated	Han et al. (2021)
piRNA-17560	FTO	BC	Erasers	oncogene	Upregulated	Ou et al. (2022)

Abbreviation:AML, acute myeloid leukemia; CRC, colorectal cancer; LC, lung cancer; ccRCC, clear cell renal cell carcinoma; HCC, hepatocellular carcinoma; EOC, epithelial ovarian cancer; NSCLC, non-small cell lung cancer; BCa, bladder cancer; PCa, prostate cancer; CC, cervical cancer; DLBCL, diffuse large B-cell lymphoma, BC, breast cancer.

## Biomarkers of ncRNAs and m^6^A regulators regarding m^6^A modifications in cancer

**FIGURE 3:**
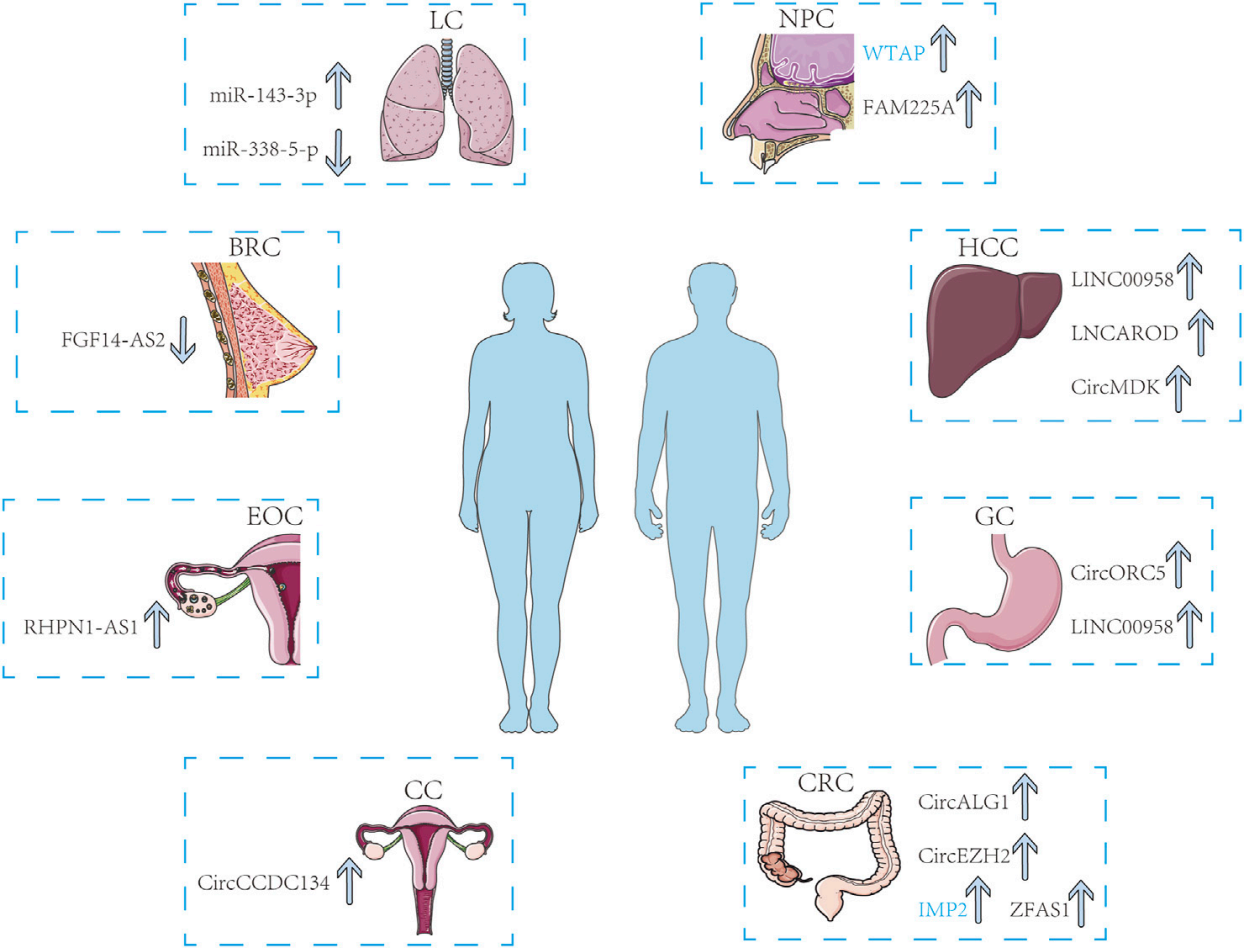
M^6^A-related ncRNAs and m^6^A regulators can be recognized as biomarkers in multiple cancers including LC, NPC, HCC, GC, CRC, CC, EOC, and BRC. The arrows show the changes in biomarkers as cancer progresses.

Multiple research projects have proven that m^6^A methylation occurs frequently in tumors, and ncRNAs modified by m^6^A and m^6^A-associated proteins have the potential to be applied as biomarkers for the diagnosis and prognosis of cancer. In this part, we summarized some common biomarkers from the respiratory system, digestive system, and reproductive system for clinical diagnosis and prognosis (Figure 3).

### Respiratory system

According to the latest studies, the stability of ncRNAs modified by m^6^A and m^6^A regulators is crucial to the advancement of respiratory cancers. As a result of WTAP m^6^A-dependent maintenance of lncRNA DIAPH1-AS1 stabilization in nasopharyngeal cancer (NPC), NPC development and metastasis are eventually aided. This research has identified WTAP as a possible biomarker for nasopharyngeal cancer and emphasizes the function of WTAP-mediated m^6^A modification in nasopharyngeal carcinoma (Li et al., 2022b). Zi-Qi Zheng et al. also discovered that FAM225A was substantially linked to survival in NPC. M^6^A was enormously concentrated in FAM225A, which boosted the long-term sustainability of its RNA and eventually promoted tumorigenesis and metastasis in NPC. We now have a new prognostic indication thanks to the clinical importance of FAM225 in NPC (Zheng et al., 2019).

In lung cancer, it has been shown that METTL3 can boost progenitor miR-143-3p splicing to support its biosynthesis and that m^6^A may stimulate progenitor miR-143-3p splicing to facilitate the production of mature miRNAs. While the miR-143-3p can indicate a poorer prognostic element for LC progression along with overall survival *in vivo*, this provides us with a predictive marker for LC metastasis (Wang et al., 2019a). Interestingly, not only m^6^A writers can regulate miRNAs, but miRNAs can also regulate m^6^A writers in turn. According to Hongyu Wu et al., overexpression of METTL3 decreased the blocking effect of miR-338-5p upon LC metastasis. MiR-338-5p was observed to suppress METTL3 production. It indicates that miR-338-5p offers a novel regulatory pathway for lung cancer and may possibly serve as a biomarker for its detection and prognosis (Wu et al., 2021b).

In conclusion, these findings reveal the roles of ncRNAs modified by m^6^A and m^6^A regulators in the development of respiratory cancers and herald their potential as predictive markers for nasopharyngeal and lung cancers.

### Digestive system

Endogenous competitive mechanisms of circRNAs and lncRNAs are strongly linked to cancer cell growth and migration in malignancies of the digestive system. In colorectal cancer, circALG1 was abundantly expressed in the tumor tissues and peripheral blood of CRC patients, and it was intimately correlated with the spread of cancer cells. Additionally, the ability of circALG1 to attach to miR-342-5p was improved by m^6^A modification, enhancing the capacity of circALG1 as a ceRNA. This shows that circALG1 could function as a prognostic indicator in CRC (Lin et al., 2022b). Similar to this, both *in vitro* and *in vivo*, circEZH2 could foster the growth and motility of CRC cells. A crucial function for circEZH2 in controlling the advancement of CRC has been indicated by the mechanism by which it may serve as a sponge for miR-133b, upregulating IGF2BP2. CircEZH2 could be another useful diagnostic marker for CRC (Yao et al., 2022). In addition, Senxu Lu et al. found significant overexpression of IMP2 and ZFAS1 in CRC cells elevated m^6^A levels. These clues may be employed independently of other biomarkers to forecast the prognosis of CRC (Lu et al., 2021). There is no denying that m^6^A modification is fundamental to CRC formation and advancement (Jiang et al., 2023). On top of that, the mutual interaction mechanism between circRNAs and lncRNAs make them promising biomarkers in CRC.

In hepatocellular carcinoma, it was shown that METTL3-mediated N6-methyladenosine modification leads to upregulation of LINC00958, which uptakes miR3619-5p and thus upregulates HDGF expression, which facilitates adipogenesis and advancement of HCC. LINC00958 plays a crucial role and highlights its value as a prognostic predictor of HCC (Zuo et al., 2020). Similar to this, it was shown that HCC patients had considerably higher expression levels of LNCAROD modified by m^6^A, which advocates HCC cell proliferation, migration, invasion, and chemoresistance. LNCAROD offers a hopeful diagnostic marker for those with HCC (Jia et al., 2021). Similarly, HCC patients’ poor survival was linked to overexpression of circMDK and modification of m^6^A. CircMDK might function as a malignant biomarker that accelerates the development of HCC (Du et al., 2022).

In gastric cancer, Hui-Ning Fan et al. discovered that circORC5 are able to take up miR-30c-2-3p while the m^6^A modification of circORC5 suppresses GC advancement by controlling the miR-30c-2-3p, suggesting that circORC5 may provide a guaranteed prognostic factor for GC (Fan et al., 2022). In addition to this, it has been asserted that LINC00958 promotes aerobic glycolysis in GC and that KIAA1429 monitors the enrichment of LINC00958 in a way that link to m^6^A modification. This shows that LINC00958 is a crucial neoplasms-promoting inducer of GC and it may be exploited as a predictive marker for clinical examination and prognostic assessment (Yang et al., 2021a).

Taken together, these reports illustrate the involvement of ncRNAs modified by m^6^A in the pathological process of digestive system tumors. Some ncRNAs modified by m^6^A can be used as diagnostic and predictive factors for digestive system tumors, which can made patients more adequately for timely treatment.

### Reproductive system

Related evidence suggests that tumor-malignant behaviors in the reproductive system are connected to fluctuations in the production of ncRNAs modified by m^6^A (Huang et al., 2022). In breast cancer, FGF14-AS2 is downregulated in an m^6^A-dependent manner through YTHDF2-mediated RNA degradation. Patients who had diminished FGF14-AS2 expression exhibited poorer DMFS, according to clinical data. As a result, it is possible that FGF14-AS2 might be employed as an indicator for bone metastasis in breast cancer. It seems to possess a substantial function in osteolysis metastasis (Zhang et al., 2022b). In contrast, circCCDC134 gets elevated in cervical cancer as a consequence of m^6^A methylation, which enhances its durability through YTHDF2. CircCCDC134 stimulates growth and dissemination of CC cells, indicating that it may be an innovative possible biomarker. It also offers new regulatory model insights for exploring the oncogenic mechanisms of circCCDC134 in CC(90). Besides, in ovarian cancer, N6-methyladenosine increased the longevity of RHPN1-AS1 methylated transcripts by hampering its deterioration, leading to the elevation of RHPN1-AS1. Furthermore, the increase of RHPN1-AS1 facilitated in the growth and spread of EOC cells. This study provides a promising prognostic indicator for EOC treatment (Wang et al., 2020b).

The above studies help us to understand the pathological process and biological changes of germline tumors from the perspective of m^6^A-related ncRNAs, and provide the m^6^A-related references of diagnostic and prognostic markers in reproductive system tumors.

## Methods for detecting m^6^A sites

With the in-depth study of tumor genesis and progression, more and more non-coding RNAs associated with m^6^A modifications have surfaced. Therefore, exploring the specific modification sites of non-coding RNAs is indispensable for the revelation of concrete regulatory mechanisms of m^6^A, the testing of biomarker utility, and the development of therapeutic approaches. For example, it has been shown that the methylation site (A783) is crucial for lncRNA HOTAIR to actuate the proliferation and aggression of breast cancer cells. Methylated A783 promotes chromatin binding of HOTAIR, breast cancer growth and migration, and gene silencing via interactions with YTHDC1 (Porman et al., 2022). Fortunately, the development of a series of m^6^A experimental assays in recent years has further advanced m^6^A-related research (Table 3).

**TABLE 3:** Experimental methods for detecting m^6^A sites.

Method	Assortment	Advantage	Disadvantage	References
MeRIP	Antibody-dependent	Boost the development of research on m^6^A	Low resolution and cannot reflect the precise location of m^6^A sites	Dominissini et al. (2015)
PA-m^6^A-seq	Antibody-dependent	Increase the resolution around the m^6^A modification site to 30 nt by 4SU-enhanced cross-linking	Merely detect m^6^A-modified sites around the 4SU operator site	Chen et al. (2015)
miCLIP	Antibody-dependent	Couple immunoprecipitation and high-throughput sequencing to accurately test for m^6^A remnants with single-base resolution	The quantity of m^6^A sites recognized by miCLIP was restricted owing to the low cross-linking efficiency	Linder et al. (2015)
m^6^A-LAIC-seq	Antibody-dependent	Measure m^6^A at the transcriptome scale	Mainly used to differentiate between methylated and unmethylated transcripts	Molinie et al. (2016)
m^6^A-REF-seq	Antibody-independent	Quantize m^6^A levels with one-base resolution	Only recognize particular m^6^A motifs (ACA), and thus its detection efficiency is poor	Bezrukov et al. (2021)
DART-seq	Antibody-independent	Separate thousands of m^6^A sites in whole RNA and characterize the quantitative m^6^A accumulation in cells	The bonding capacity between the integrated APOBEC1-YTH protein and the m^6^A modification may influence the testing precision of the target	Meyer (2019)
m^6^A-label-seq	Antibody-independent	Detect m^6^A at a monobase resolution level by metabolically tag the candidate substrate adenosine during m^6^A production	Only be applied to cellular systems	Shu et al. (2020)
m^6^A -SEAL	Antibody-independent	Higher sensitivity and specificity	Have not yet reached the condition of single-base resolution	Wang et al. (2020c)

### Antibody-dependent methods

In the early days of research, the methods for detecting m^6^A sites were almost exclusively antibody-dependent, and this has played an essential role up to now. Methylated RNA immunoprecipitation sequencing (MeRIP), the earliest m^6^A detection method, is a combination of m^6^A-methylated mRNA segments and high-throughput sequencing, which can detect the m^6^A-modified areas of approximately 100 nt–200 nt (Dominissini et al., 2015). The emergence of MeRIP has tremendously boosted the development of research on m^6^A. However, it is undeniable that the method has low resolution and cannot reflect the precise location of m^6^A sites.

With the rapid advancement of science and technology, cross-linking and immunoprecipitation (CLIP), another antibody-dependent method for detecting the m^6^A locus, is becoming well-known and widely used. Through engagement with ultraviolet (UV) irradiation, CLIP establishes covalent bonds between proteins and RNA, which can stabilize protein-RNA interactions. Photo-cross-linking-assisted m^6^A sequencing strategy (PA-m^6^A-Seq), m^6^A individual-nucleotide resolution UV crosslinking and immunoprecipitation (miCLIP), and m^6^A-level and isoform-characterization sequencing (m^6^A-LAICseq) are all associated with CLIP. PA-m^6^A-seq can increase the resolution around the m^6^A modification site to 30 nt by 4SU-enhanced cross-linking (Chen et al., 2015). Nevertheless, PA-m^6^A-seq can merely detect m^6^A-modified sites around the 4SU operator site; moreover, due to the metabolism of 4SU, PA-m^6^A-seq seems to be only available for use in cells. Overcoming some of the drawbacks of PA-m^6^A-seq, miCLIP couples immunoprecipitation and high-throughput sequencing to accurately test for m^6^A remnants with single-base resolution (Linder et al., 2015). However, the quantity of m^6^A sites recognized by miCLIP was restricted owing to the low cross-linking efficiency. Comparatively, m^6^A-LAIC-seq measures m^6^A at the transcriptome scale and is mainly used to differentiate between methylated and unmethylated transcripts (Molinie et al., 2016).

### Antibody-independent methods

In recent years, antibody-independent methods have gained popularity in the search for more effective m^6^A site detection methods. For instance, MazF is an m^6^A-sensitive ribonucleic acid endonuclease that can cut ACA sequences but not m^6^ACA sequences. MAZTER-seq and m^6^A- REF-seq were created because of this attribute (Bezrukov et al., 2021). Although m^6^A-REF-seq can quantize m^6^A levels with one-base resolution, it only recognizes particular m^6^A motifs (ACA), and thus its detection efficiency is poor.

Another antibody-free technique called DART-seq uses a fusion constructed from APOBEC1 (cytidine deaminase) and the YTH structural domain to detect the m^6^A site by inducing C-to-U deamidation at a site adjacent to the m^6^A residue. DART-seq can separate thousands of m^6^A sites in whole RNA and characterize the quantitative m^6^A accumulation in cells (Meyer, 2019). Obviously, the bonding capacity between the integrated APOBEC1-YTH protein and the m^6^A modification may influence the testing precision of the target.

Besides, m^6^A-label-seq is a metabolic labeling approach. It detects m^6^A at a monobase resolution level by metabolically tagging the candidate substrate adenosine during m^6^A production (Shu et al., 2020). Unfortunately, this method can only be applied to cellular systems.

The FTO-assisted m^6^A selective chemical labeling method, also known as m^6^A -SEAL, is also an innovative technique. In the presence of dithiothreitol (DTT)-mediated thiol addition reactions, unstable N6-hydroxymethyladenosine (hm^6^A) can be converted to ultrastable N6-dithiocarbonylmethyladenosine (dm^6^A). And m^6^A-SEAL is associated with the role of hm^6^A in RNA that can be oxidized by the FTO enzyme of m^6^A, which can effectively detect m^6^A modification sites (Wang et al., 2020c). Compared to the previous technique, m^6^A-SEAL has higher sensitivity and specificity; however, this technique has not yet reached the condition of single-base resolution.

So far, although there are numerous methods for the detection of the m^6^A sites, they all have their own defective aspects. Therefore, only through the comprehensive utilization of different detection methods and the pursuit of more efficient and accurate m^6^A site detection approaches can we further boost the progress of m^6^A-related studies.

## Conclusion and perspective

In this review, we concluded, on the one hand, the role that m^6^A regulators play in tumorigenesis and development by modulating non-coding RNAs. On the other hand, we also outlined the ability of non-coding RNAs to influence m^6^A modifications that promote or attenuate malignant changes in cancer. In addition, we summarized some non-coding RNAs and m^6^A-related proteins that may be employed as diagnostic and prognostic indicators in the hopes that this information will help clinical practice. Considering the practical application of biomarkers, we also aggregated some useful m^6^A site detection methods in recent years, which can be helpful for researchers to better choose the experimental methods.

The epigenetic processes of cancer cells have undergone fundamental changes (López et al., 2022). The m^6^A modification of RNAs, one of the epigenetic research hotspots, is crucial for the regulation of cancer. Multiple researches have proven that m^6^A-related proteins have the ability to control the production and biological functions of ncRNAs by altering their methylation levels. In cancer, this can manifest as tumor growth, metastasis, and stem cell differentiation, among others. Some progress has been made in the study of the biological effects of m^6^A on tumors, but there are still some limitations that require further breakthroughs. Currently, the gene knock-out of tissue-specific m^6^A-related proteins in mice can help reveal the mechanisms of m^6^A modification in related cancer cells. However, we still need more clinical trials to measure the diagnostic value and therapeutic effects of m^6^A modification on patients. With the development of more innovative high-end research tools and research methodologies based on m^6^A, we believe that in the near future we can conduct more basic and clinical studies to fill the gap in current research (Meng et al., 2023).

NcRNAs may control the gene expression level and function of m^6^A-related proteins as well. This may be related to the influence of ncRNAs on their transcription, translation, and protein capacity of m^6^A regulators. Correlations between m^6^A modifications and ncRNAs offer a fresh route for us to explore precise processes underlying the emergence of cancer. Some studies, however, were only able to analyze publicly accessible datasets statistically. The non-coding RNAs’ capacity to predict cancer continues to be constrained due to the comparatively small number of statistical samples (Mo et al., 2021).

With the gradual increase in the importance of m^6^A modification in the medical community, abnormally expressed m^6^A-related non-coding RNAs have recently been identified in peripheral bloodstreams of patients who suffer from cancers, which tremendously improves the convenience and utility of m^6^A-modified non-coding RNAs as biomarkers with good prospects for clinical application (Eun et al., 2023). In addition, cancer has inappropriate expression of m^6^A-related proteins, which accordingly affects the m^6^A level of ncRNAs. Taking into account the views expressed above, it is reasonable to assume that m^6^A-related proteins and m^6^A-modified non-coding RNAs may be exploited as promising clinical diagnostic and prognostic markers. However, current studies on m^6^A-related ncRNAs and m^6^A regulators are still incomplete, and the proposed potential m^6^A-related diagnostic and prognostic markers still need to be validated by more advanced m^6^A site detection methods.

## Abbreviations

m^6^A, N6-methyladenosine; METTL3, methyltransferase-like 3; METTL14, methyltransferase-like 14; WTAP, Wilms tumor 1-associating protein; KIAA1429, vir-like m6A methyltransferase-associated protein; RBM15/15B, RNA-binding motif protein 15/15B; ZC3H13, zinc finger CCCH structural domain-containing protein 13; FTO, fat mass and obesity-associated protein; ALKBH5, alkylation repair homolog protein 5; YTHDF, YT521-B homology domain family; YTHDC, YT521-B homology domain-containing family; HNRNPC, heterogeneous nuclear ribonucleoprotein C; IGF2BP, insulin-like growth factor 2 binding proteins 2; mRNA, messenger RNA; miRNA, microRNA; lncRNA, long noncoding RNA; circRNA, circular RNA; piRNA, piwi-interacting RNA.
